# Composite Sulfonated Polyether-Ether Ketone Membranes with SBA-15 for Electrochemical Energy Systems

**DOI:** 10.3390/ma13071570

**Published:** 2020-03-29

**Authors:** A. Rico-Zavala, J. L. Pineda-Delgado, A. Carbone, A. Saccà, E. Passalacqua, M.P. Gurrola, A. Alvarez, S. Rivas, J. Ledesma-García, L.G. Arriaga

**Affiliations:** 1 Centro de Investigación y Desarrollo Tecnológico en Electroquímica S.C., Sanfandila 76703, Mexico; arico@cideteq.mx (A.R.-Z.); jpineda@cideteq.mx (J.L.P.-D.); 2 CNR-ITAE, Institute for Advanced Energy Technologies “N. Giordano”, 98126 Messina, Italy; carbone@itae.cnr.it (A.C.); sacca@itae.cnr.it (A.S.); enza.passalacqua@itae.cnr.it (E.P.); 3 Cátedra Consejo Nacional de Ciencia y Tecnología-Tecnológico Nacional de México/ Instituto Tecnológico de Chetumal, Chetumal 77013, Mexico; polett@itchetumal.edu.mx; 4 Tecnológico Nacional de México/ Instituto Tecnológico de Chetumal, Chetumal 77013, Mexico; 5 Facultad de Ingeniería, División de Investigación y Posgrado, Universidad Autónoma de Querétaro, Querétaro 76010, Mexico; alejandra.alvarez@uaq.mx (A.A.); janet.ledesma@uaq.mx (J.L.-G.)

**Keywords:** S-PEEK composite membrane, PEMFC, electrochemical hydrogen compression

## Abstract

The aim of this work is the evaluation of a Sulfonated Poly Ether-Ether Ketone (S-PEEK) polymer modified by the addition of pure Santa Barbara Amorphous-15 (SBA-15, mesoporous silica) and SBA-15 previously impregnated with phosphotungstic acid (PWA) fillers (PWA/SBA-15) in order to prepare composite membranes as an alternative to conventional Nafion^®^ membranes. This component is intended to be used as an electrolyte in electrochemical energy systems such as hydrogen and methanol Proton Exchange Membrane Fuel Cell (PEMFC) and Electrochemical Hydrogen Pumping (EHP). The common requirements for all the applications are high proton conductivity, thermomechanical stability, and fuel and oxidant impermeability. The morphology of the composite membranes was investigated by Scanning Electron Microscopy- Energy Dispersive X-ray Spectroscopy (SEM-EDS) analysis. Water Uptake (Wup), Ion Exchange Capacity (IEC), proton conductivity, methanol permeability and other physicochemical properties were evaluated. In PEMFC tests, the S-PEEK membrane with a 10 wt.% SBA-15 loading showed the highest performance. For EHP, the inclusion of inorganic materials led to a back-diffusion, limiting the compression capacity. Concerning methanol permeability, the lowest methanol crossover corresponded to the composites containing 5 wt.% and 10 wt.% SBA-15.

## 1. Introduction

Currently, there is a problem in the energy sector that affects the global environment. Problems related to energy supply and use are related not only to global warming, but also to additional issues such as air and water pollution, acid precipitation, ozone layer degradation, deforestation and emission of radioactive substances [1]. In addition, global energy demand continues to increase and an increase of up to an order of magnitude is expected by 2050. Therefore, the search for new technologies for sustainable energy generation will become more important over time. Therefore, in order to reduce these problems, several alternatives have been proposed. Electrochemical devices, Proton Exchange Membrane Fuel Cell (PEMFC), Direct Methanol Fuel Cell (DMFC), and Electrochemical Hydrogen Pumping or Compression (EHP or EHC) are recognized as sources of clean and sustainable energy production [2].

PEMFCs are considered very efficient devices for clean energy production, since only water and heat are generated as by-products. On the other hand, EHP is a recent alternative for hydrogen purification, pumping, and storage [3,4]. An EHP has the same components of a PEMFC, but the operation is different: in place of a power generator, the device is used to compress hydrogen from anode to cathode, where the H^+^ ions produced at the anode are reduced to form H_2_. The required energy for this process is low, since a 0.3 V potential pulse is enough to promote H_2_ oxidation on one side of the compressor and reduction in the pumping chamber [5,6]. It is evident that in this system, the membrane is a crucial component and proton conducting membranes capable to withstand high pressure difference between anode and cathode sides are required. All these devices use a Membrane-Electrode Assembly (MEA) as the main active component, where the membrane has the function of electrolyte, which must have high proton conductivity, high thermal and mechanical resistance and low or no permeability to fuels [7]. The current PEMFC and EHP technology utilizes Nafion^®^ as an electrolyte, a perfluorosulfonic acid (PFSA) polymer membrane, whose proton conductivity is highly influenced by the amount of water present in the membrane, and it is optimal when the membrane is fully saturated with water. Operating PEMFCs under low humidity (under 100%) or high temperature (over 80 °C) leads to membrane dehydration, which remarkably reduces the proton conductivity [8,9]. It is thus highly desirable to explore alternative polymers sometimes modified with appropriate hydrophilic inorganic fillers able to be competitive with the Nafion^®^, from the point of view of proton [10].

In this sense, S-PEEK membranes appear as an alternative to PFSA (perfluorosulfonic acid, like Nafion^®^) for PEMFC due to their low cost and thermomechanical stability [11]. S-PEEK is the result of the sulfonation reaction of Poly Ether-Ether Ketone (PEEK), a thermoplastic polymer which can only be chemically modified by dissolution in concentrated sulfuric acid or chlorosulfonic acid [12]. This reaction is not easy to control, when a 100% sulfonation degree (SD) is reached, the resulting polymer is water soluble, above 70% is soluble in methanol and S-PEEK membranes with a SD > 60% are considerably deformed in water and methanol-water solution at 80–90 °C, below 50% are soluble in organic solvents such as dimethylacetamide (DMAc), dimethylformamide (DMF) and dimethylsulfoxide (DMSO) [13]. Despite the good properties of S-PEEK membranes, strategies for further improvement have been sought. For example, to decrease liquid or gas fuel permeability and to provide mechanical stability, the inclusion of hydrophilic inorganic materials is possible to improve the water retention of the membranes, limiting the swelling and solid acids, such as heteropolyacids (HPAs) as phosphotungstic acid PWA [14]. HPAs are considered proton-conducting super acids, and are a subset of polyoxomethalates, which contain a central heteroatom, additional atoms, oxygen and hydrogen. HPAs are one of the most attractive inorganic modifiers since, due to their crystalline structure, are highly conductive and thermally stable [15]. In this last case, it has been found that a way to carry out this modification is to immobilize the HPA on a high surface area material, to reduce the HPA elution [16,17,18]. Other modifications already published for other authors as A. Filippov et al., studied the effect of transport asymmetry in perfluorinated membranes previously modified by 4% halloysite nanotubes (HNT) with platinum loading; they development a theory that can predict the transport properties of these membranes [19]. Further, the authors evaluated the PEMFC performance and the increase in the specific power due to a membrane self-humectation. This phenomenon is attributed to the interaction between oxygen and hydrogen with the Pt/HNT in the membrane bulk [20].

In this paper, an investigation on MEAs based on S-PEEK composite membranes was carried out to verify their applicability in different electrochemical devices such as PEMFC and EHP. Membranes based on sulfonated PEEK with 50% sulfonation degree and different loadings of SBA-15 (5–15 wt.%) were prepared and characterized in terms of physico-chemical measurements and electrochemical tests. Moreover, the influence of the introduction of HPA in SBA-15 was also evaluated.

## 2. Experimental

### 2.1. Synthesis of the Inorganic Filler

Silica material SBA-15 was synthesized using the sol-gel method under acid conditions. The copolymer Pluronic 123 (BASF) dissolved in 4 M HCl was used as the structure directing agent. Tetraethyl-orthosilicate (TEOS 98%, Sigma Aldrich) was added to the solution and kept at 35 °C under constant stirring for 24 h. This solution was then transferred to a polypropylene bottle and heated at 80 °C for 24 h. Successively, the obtained solid was filtered and washed with deionized water, then dried at room temperature for at least 24 h. This powder was dried at 110 °C for 18 h and calcined at 500 °C for 5 h to remove the surfactant.

PWA (Phosphotungstic acid, H_3_PW_12_O_40_, Sigma Aldrich) was supported on SBA-15 (3:7 wt.%) by impregnation in water. After PWA was completely dissolved in distilled water, the SBA-15 was added and kept under ultrasonic mixing for 3 h. Water was evaporated at 80 °C and the powder dried at 100 °C for 2 h.

### 2.2. Sulfonation of the Poly-Ether-Ether-Ketone (PEEK)

Sulfonated PEEK (S-PEEK) was prepared by reaction of 5 g of PEEK (PEEK Victrex^®^ powder grade PF450) in 100 mL of concentrated sulphuric acid (H_2_SO_4_ from J.T. Baker) at 30 °C for 24 h under constant stirring in order to obtain a 50% sulfonation degree [21]. The polymer was precipitated in cold water, washed with deionized water until a neutral pH of rinse water was reached. The sulfonated polymer was dried over night at 70 °C, followed by 2 h at 120 °C.

### 2.3. Membranes Preparation

The membranes were prepared following a standardized casting procedure as reported elsewhere [21]. The composite membranes were prepared adding the SBA-15 or PWA/SBA-15 after the polymer was completely dissolved in dimethylformamide (DMF from Sigma Alcrich). Different SBA-15: S-PEEK ratios (wt.%) were used (5:95, 10:90, 15:85), the PWA/SBA-15 membrane was prepared considering a SBA-15: S-PEEK ratio of 10:90.The membranes were obtained for evaporation of solvent at 80 °C for 3 h, then were chemically treated in 1M H_2_SO_4_ for 2 h at 80 °C and soaked in deionized water at the same temperature for 1 h. Membrane thickness was 70–95 μm.

### 2.4. MEAs Fabrication

The MEAs were prepared by cold pressing the gas diffusion electrodes (GDEs) to the membrane. GDEs were obtained by hot-spray deposition of the catalyst ink over a 5 cm^2^ SIGRACET^®^ 35BC gas diffuser. The catalyst layer for anode and cathode consisted in a 20 wt.% Pt on carbon (Vulcan XC-72) with 0.9 mg cm^−2^ Pt loading on each GDE.

### 2.5. Inorganic Filler and Membranes Characterization

#### 2.5.1. Inorganic Filler Characterization

The textural properties of the inorganic fillers were analyzed by means of adsorption–desorption isotherms of N_2_ at −196.15 °C using a Micromeritics TriStar 3000. The surface area was calculated according to the Brunauer–Emmett–Teller (BET) equation. The fillers were also characterized by X-ray Diffraction performed by a Philips X-ray automated diffractometer (model PW3710) with Cu Kα radiation source. The 2θ Bragg angles were scanned between 5° and 100° 2θ. SBA-15 filler morphology was investigated by Scanning Electron Microscopy (SEM using a JEOL model JSM-6060 LV microscope) and High-Resolution Transmission Electron Microscope (HRTEM JEOL JEM-2000FX FASTEM).

#### 2.5.2. Composite Membranes Morphology

Composite membrane morphologies were investigated by scanning electron microscopy and energy dispersive spectroscopy (SEM-EDS using a JEOL model JSM-6060 LV microscope). The samples were dried in oven vacuum at 80 °C, then all membranes were coated with carbon for improving the SEM-EDS analysis. Elements mapped were carbon, oxygen, sulphur, silicon, and tungsten.

#### 2.5.3. Water uptake (W_up_), Swelling, Ion Exchange Capacity (IEC), Proton Conductivity ($\sigma_{H^{+}}$) and Methanol Permeability

Water uptake (W_up_%) was determined according to the following Equation:

(1) $$W_{\mathrm{Up}}=\left(\frac{m_{\mathrm{wet}}-m_{\mathrm{dry}}}{m_{\mathrm{dry}}}\right)\;\times \;100$$

where m_wet_ and m_dry_ are the wet and dry weights of the membranes. Thickness, area and weight of each membrane were measured after being dried in a vacuum oven at 80 °C for 2 h and after soaking the membrane in distilled water at room temperature (24 h).

Swelling is calculated by V_wet_/V_dry_, being the wet and dry volume of the membranes at the corresponding conditions.

IEC was determined through an acid-base titration with an automatic titrator Metrohm (751 GPD Titrino) on samples previously dried at 80 °C for 2 h under vacuum (1000 mbar). The membrane was soaked in 1 M NaCl solution to exchange the H^+^ of the SO_3_H groups with Na^+^, and as a titrant a 0.01 M NaOH solution was used. The IEC values were calculated using the following Equation:

(2) $$\mathrm{IEC}=\frac{V\cdot M}{m_{\mathrm{dry}}}$$

where V_NaOH_ (mL) is the added titrant volume at the equivalent point; M, is the molar concentration of the titrant, m_dry_ is the dry membrane mass and IEC is the ionic exchange capacity (SO_3_H meq/g).

Proton conductivity was determined using the four-point method in the longitudinal direction in a commercial conductivity cell (Bekktech). The cell was kept immersed in deionized water and connected to a PGSTAT Autolab 302 Potentiostat/Galvanostat equipped with a booster of 20 A (Metrohm^®^). The conductivity was determined from 30 °C up to 80 °C with the following Equation [22]:

(3) $$\sigma_{H^{+}}=\frac{L}{R\cdot W\cdot T}$$

where $\sigma_{H^{+}}$ is the proton conductivity of the sample (S cm^−1^); ***L*** is the distance between the Pt electrodes determined by the cell design (0.425 cm); ***R*** is the polymer resistance (Ω); and W and T are the width and thickness of the membrane (cm), respectively.

The λ value (expresses as moles H_2_O/moles-SO_3_H) was calculated through the water uptake and IEC values ratio, both expressed in moles. To determine the number of the water molecules per suphonated group, λ, Equation (4) was used, where 18 is the molar mass of water [23]:

(4) $$\lambda =\frac{W_{\mathrm{Up}}\cdot 10}{18\cdot \mathrm{IEC}}$$

Methanol permeability was investigated with a Potentiostat/Galvanostat Bio-logic^®^ by using a two-compartment diffusion cell. Compartment I (C_I_) was filled with solution 1 M methanol in 0.5 M H_2_SO_4_, compartment II (C_II_) contains 0.5 M H_2_SO_4_ solution. The methanol concentration in C_II_ was monitored by chronoamperometry technique applying a constant potential of 0.68 V vs Ag/AgCl, lasting 60 s each hour. A Pt micro-electrode was used as working electrode, Ag/AgCl as reference, and Pt wire as counter electrode [24]. The methanol permeability was estimated using the following equation:

(5) $$P=D\cdot K=\frac{V_{\mathrm{II}}\cdot L\cdot C_{\mathrm{II}}}{A\cdot C_{I}\cdot t}$$

where C_I_ and C_II_ is the methanol molar concentration in the compartment I and II in mol∙L^−1^, respectively. A is the exposed geometrical area of membrane in cm^2^ and L is the membrane thickness in cm. V_II_ is the compartment II volume in cm^3^, t is the permeation time in s, D is methanol diffusivity, and K is partition coefficient between the membrane and the adjacent solution. Finally, the D∙K product corresponds to permeability in cm^2^∙s^−1^.

### 2.6. Electrochemical Characterization

#### 2.6.1. Proton Exchange Membrane Fuel Cell

A regular 5 cm^2^ PEM fuel cell by Electrochem^®^ was used to evaluate the membrane performance for PEMFC and EHC operation mode. An AUTOLAB^®^ potentiostat/galvanostat PGSTAT 302 coupled to a Booster 20 A was used for the tests (linear voltammetry, electrochemical impedance spectroscopy and chronoamperometry). Reactive gas conditions (temperature, flow, pressure and relative humidity) were managed by means of a PSCompuCell Fuel Cell Test Station by Electrochem^®^. Hydrogen and oxygen flow rates were fixed at 120 and 300 ml min^−1^ respectively supplied to the FC at 40 psi, 30 °C (100% RH) without back pressure control.

Linear voltammetry (I-V curve) was performed from open circuit potential to 0.2 V at 50 mV s^−1^ scan rate. Electrochemical impedance spectroscopy was performed at open circuit potential, in a frequency range from 100 kHz to 0.1 Hz, to determine the ohmic resistance that accounts for the resistance due to ionic and electronic current in the electrolyte and in the electrodes respectively.

#### 2.6.2. Electrochemical Hydrogen Pumping

The hydrogen was supplied fully humidified to the EHC at 120 ml min^−1^ flow rate. The pumping capacity of each membrane was evaluated by chronoamperometry with potential pulses from 100 to 700 mV for 10 min each. A back-pressure regulator (BPR) was used to follow the pressure increment in the pumping chamber, while the potential pulse was applied. According to the Nernst equation, a thermodynamic cell voltage of 30 mV would be required at 298 K for 10 P_cathode_/P_anode_ ratio. Since hydrogen oxidation and evolution reactions are highly reversible, the greater polarization contribution comes from ohmic losses, therefore typical cell voltage is from 50 to 100 mV [3]. Higher voltage pulses were applied in this work, in order to find the mass transport limiting conditions for each membrane, since its efficiency as a compressor is highly dependent on the hydration conditions [25].

## 3. Results

### 3.1. Filler Characterization

**Figure 1:**
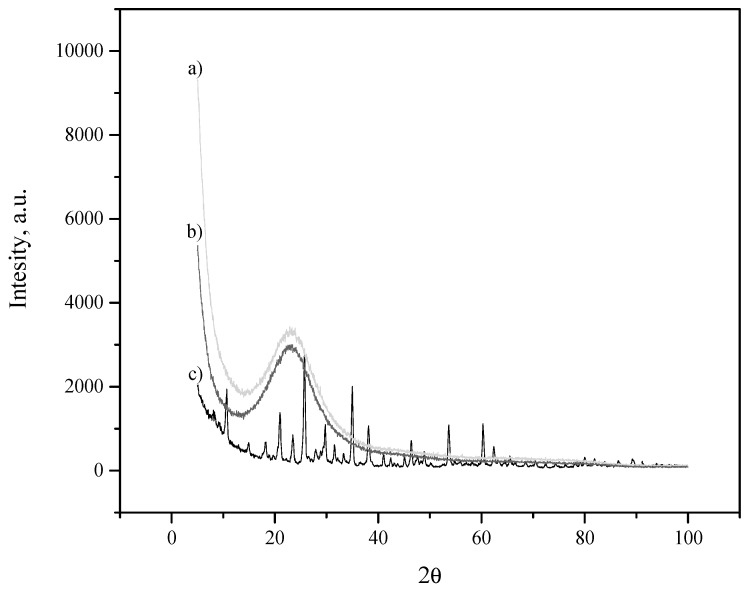
XRD patterns of the fillers (**a**) SBA-15, (**b**) PWA/SBA-15, (**c**) PWA.

The X-ray diffraction patterns of the fillers are shown in Figure 1. The PWA diffraction pattern corresponds to the crystal structure, elsewhere reported [26]. The SBA-15 and PWA/SBA-15 have a broad reflection at about 2θ = 24°, characteristic of amorphous silica. The PWA characteristic peaks are absent in the diffraction pattern of PWA/SBA-15, thus, the PWA could be within the SBA-15 pores. In SiO_2_ (200–300 m^2^ g^−1^) the crystalline phase of a heteropolyacid appears only at loads higher than 20 wt.% [21,26]. In mesoporous materials such as MCM-41 silica (1250 m^2^ g^−1^), the PWA peaks are observed only at loads higher than 50 wt.%, and TEM analysis reveals that PWA is mainly located inside the MCM-41 channels [27]. Given the PWA solubility in water, it is not possible to conclude from XRD patterns if the heteropolyacid is in the SBA-15 pores.

**Figure 2:**
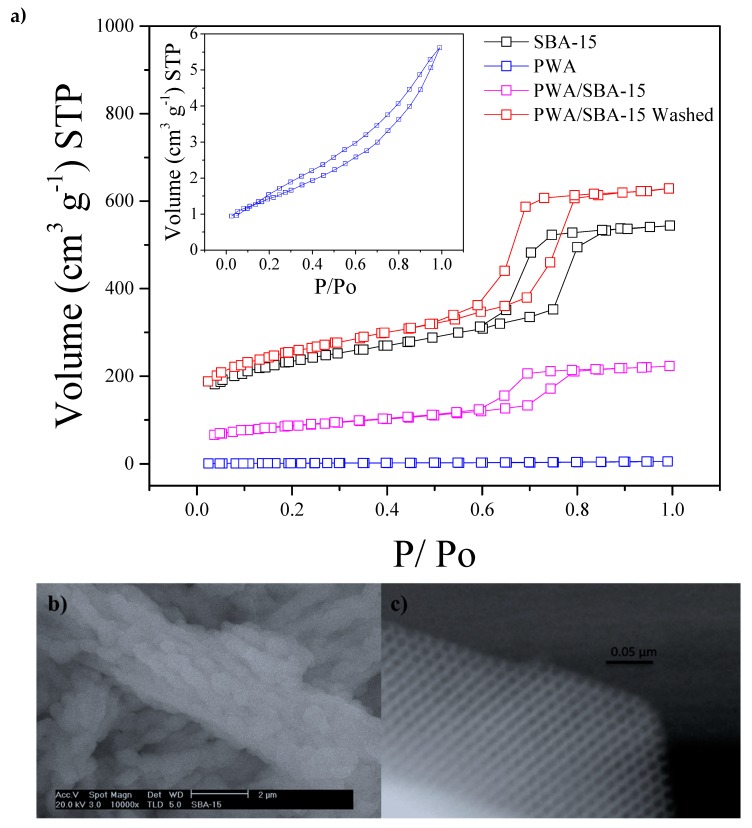
(**a**) Adsorption / desorption isotherms of inorganic fillers; (**b**) SEM morphology of SBA-15; (**c**) HRTEM image of SBA-15.

Textural properties of the fillers are shown in Table 1. As expected, SBA-15 has high surface area which decreases once the PWA is incorporated (PWA/SBA-15); heteropolyacid particles are possibly incorporated into the support matrix channels, thus clogging their pores [28]. Due to the low surface area value of the supported PWA, compared to that obtained for the SBA-15, it was decided to wash the mixture and thus observe its retention in the SBA-15. So, 0.5 g PWA/SBA-15 were dispersed under stirring in deionized water for 15 min and then filtered and dried at 80 °C overnight, textural properties where measured again PWA/SBA-15 washed. A more detailed analysis of the surface area values presented in Table 1 is completed with the N2 adsorption–desorption isotherms shown in Figure 2a. The isotherms shown for SBA-15, PWA / SBA-15 and PWA / SBA-15 washed, are type IV according to the IUPAC, which are characteristics of mesoporous solids where multilayer adsorption occurs. This type of adsorption reflects a central area of the ascending isotherm as more layers are adsorbed on solid surface. On the other hand, the sample that only contains PWA is also observed, which shows a hysteresis type II, characteristic of non-porous materials, corresponding to the small surface area value. Figure 2b shows the morphology of the synthesized materials that has been studied by Scanning electron microscopy. The image of the SBA-15 mesoporous silica shows that the material has the shape of curved cylinders with high uniformity of approximately 400–500 nm in diameter and of about 1–1.2 nm long. The image obtained by HRTEM of the mesoporous silica material is presented in the Figure 2c. The material corresponds to a highly ordered mesoporous structure with a hexagonal arrangement of uniform nanotubular pores and that it corresponds to the structure of the SBA-15 mesoporous silica. These results confirm that the pore diameter and BET surface area for the inorganic fillers are not dependent on the silica source, but they are dependent on the nature of the directing agent (Pluronic P123) and its associated cosolvent that induces the meso-structure formation with a pore diameter between 9.4 and 9.3 nm (SBA-15 and PWA/SBA-15 Washed) [24,29].

With this analysis, it is possible to show that textural properties presented for different materials are characteristics of high surface area materials except for the PWA sample [30].

After washing the sample of PWA/SBA-15, surface area and pore diameter increased close to SBA-15 values, indicating that PWA is not bound inside of the SBA-15, so it does not work as a container in water immersion conditions, and consequently PWA is diluted in water.

### 3.2. Membranes Characterization

**Figure 3:**
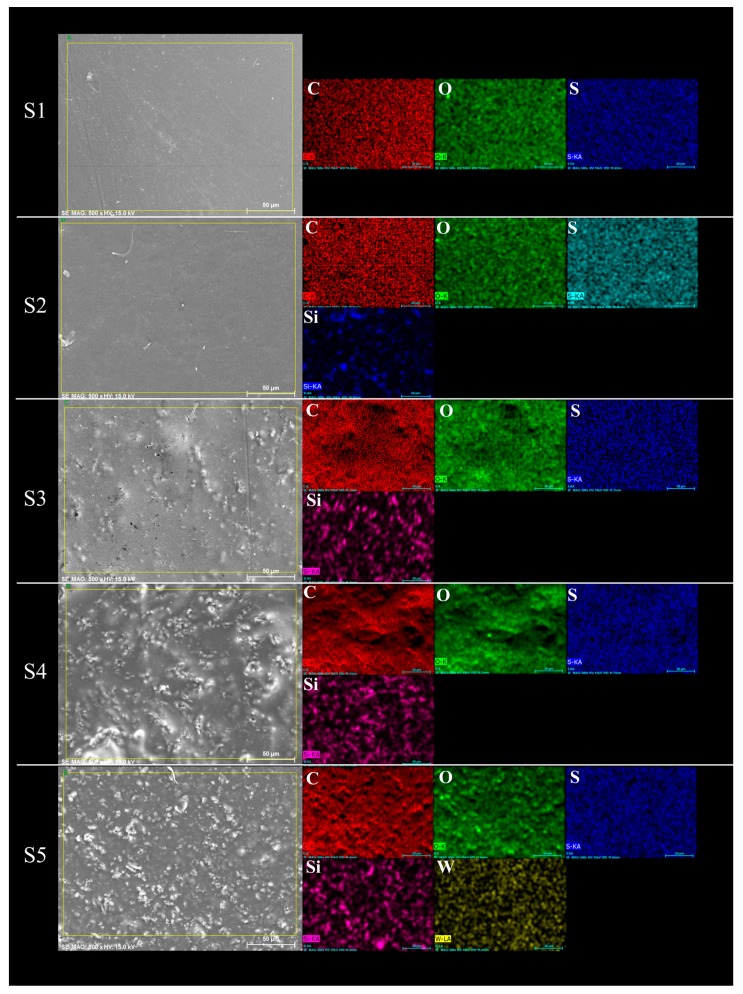
Morphologies of the different membranes investigated by SEM-EDS.

In Figure 3, the membranes morphology is shown; when the contained of inorganic filler increase, SBA-15 is agglomerated in the polymer matrix. These phenomena are confirmed by EDS analyses. This agglomeration allowed the formation of micropores in the polymer matrix, which could be detrimental to the performance of the membranes in cell tests. On the other hand, SBA-15 well dispersed could decrease the methanol permeability of membranes, forming a barrier for methanol crossover [24].

Water uptake of S-PEEK is highly dependent of its sulfonation degree (SD). A high SD means high W_up_, but also higher swelling, and therefore low mechanical stability [31]. The incorporation of hydrophilic materials such as SiO_2_ into a polymeric matrix, improve water retention capacity at high operating temperature [32] as well as mechanical stability. In Table 2, the physico-chemical parameters for the prepared membranes are reported at 30 °C and compared to pristine S-PEEK and Nafion 115 as reference.

The ionic exchange capacity of the pristine S-PEEK corresponds to 54% sulfonation degree. The addition of SBA-15 into the polymeric matrix causes a reduction of IEC values due to the presence of filler without exchangeable protons. On the contrary, the IEC of sample S5, containing PWA, increases from 1.36 (the corresponding loading in composite membrane S3) to 1.42 meq g^−1^, meaning that the heteropolyacid is incorporated into the pores of the filler. Similarly, the Wup value decreases when the minimum amount of SBA-15 is added (S2). Wup value increases by increasing the loading in composite membranes, due to the hydrophilic properties of the filler.

The swelling data confirm that the inserted fillers directly participate to the water management of the polymer membrane matrix. In fact, the swelling of 1.2 found for sample S2, with a Wup of 40 wt.%, does not correspond to the swelling of sample S5 with the same Wup value. Considering that in the swelling calculation only the geometrical parameters are considered, it can be concluded that a Wup of 40 wt.% is differently located in the two samples. In sample S2 the water is more distributed in the polymeric matrix while in S5 it is more coordinated to the filler.

The λ values are related to the capacity of sulfonic groups to coordinate water molecules and the trend is in accordance with water uptake and swelling data. In particular, the sample S3, despite the lowest IEC value, shows the highest λ and water uptake while maintaining swelling values comparable to other membranes. This sample seems to be the most promising for the electrochemical tests.

**Figure 4:**
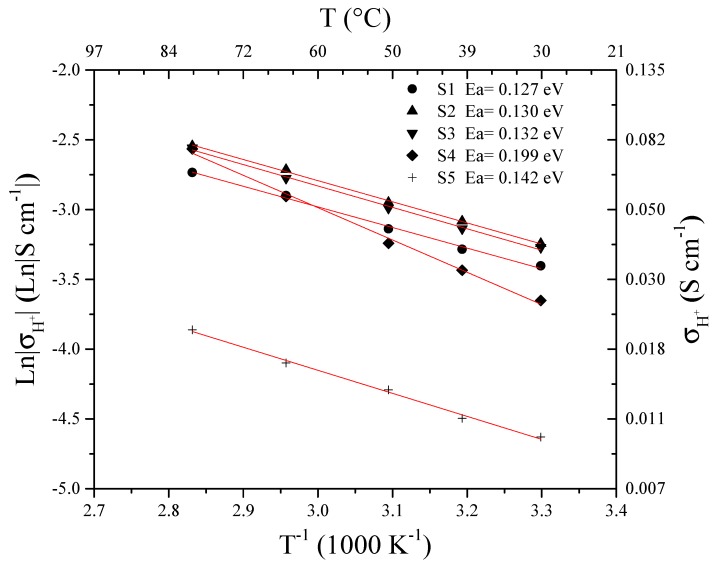
Arrhenius plot of proton conductivity as a function of temperature.

Figure 4 shows proton conductivity values as a function of the linearized temperature according to the Arrhenius Equation:

(6) $$\sigma_{H^{+}}=\sigma_{o}\cdot e^{-\left(\mathrm{Ea}/R\cdot T\right)}$$

where $\sigma_{H^{+}}$ is the proton conductivity in S cm^−1^, $\sigma_{0}$ is the pre-exponential factor in S cm^−1^, Ea is the energy activation, R is the constant of gases and T is the temperature in kelvin. From these curves, the activation energies can be obtained for all the membranes in a range of 0.127 eV to 0.199 eV indicating a high deprotonation of the sulfonic group (−SO_3_H) in addition to the predominance of the Grotthuss mechanism (carried out from 0.1 eV to 0.5 eV) for proton transport, according with [33,34]. Energies activation suggest that membrane with 15% load (S4) requires more energy to carry out the transport of protons, possibly in the high content of filler, which, at low temperatures, damages proton transport by blocking the conducting groups. In addition, this high loading adds tortuosity to the membrane [35], increasing in this way the resistance to ionic charge transfer. Meanwhile, for the membranes with 5% and 10% load, similar proton conduction behaviors are shown. On the other hand, the S5 membrane, although it exhibits slopes similar to the membranes S1, S2 and S3, is deficient for the proton conduction indicated by the low pre-exponential factor [36] (see Table 3). This low value indicates that the collisions for the protons transfer are reduced, somehow prevented, possibly because the incorporated PWA is in closed channels, with inaccessibility for the crossing of charge from one side to another, although the membrane presents good deprotonation (indicated by the activation energy, 0.142 eV) [37].

**Figure 5:**
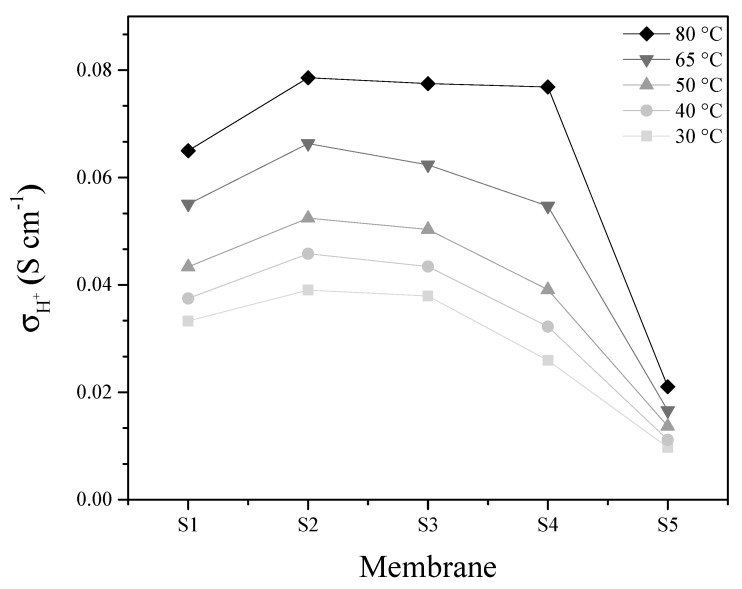
Proton conductivity at different temperatures as a function of composite polymer.

Figure 5 shows the conductivity plots at different temperatures as a function on the used membrane. S2 membrane exhibits an improved proton conductivity compared to the other membranes, possibly due to the low SBA15 loading (5%). In this membrane improves the water retention without blocking the SO_3_H groups of the polymer, meanwhile for the other membranes the inorganic excess load makes deficient the proton transport at low temperature. At 80 °C the conductivity values in the membranes are almost identical for S2, S3 and S4. These is because, at high temperature, the deswelling is increased, improving the interconnectivity of ionic channels, thus favoring the conduction of protons [11]. In the case of the membrane with PWA, the conductivity is reduced, with the incorporation of both fillers (SBA15 and PWA) possibly resulting in a low proton transport and low swelling (see Table 3).

**Figure 6:**
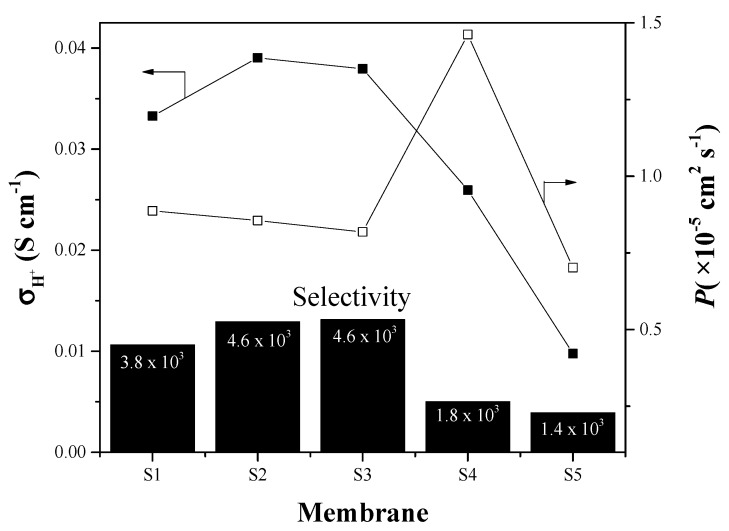
Proton conductivity, methanol permeability at 8 h and proton selectivity under wet conditions at 30 °C.

The same membranes are also considered for direct methanol fuel cell application, and in this case their capability to block the methanol cross-over is evaluated. Figure 6 shows the proton conductivity, methanol permeability at 8 h in the chamber C_II_ of the diffusion cell. It is evident that methanol permeability is higher in sample S4 than others. This behavior is due to the filler works as a barrier, blocking the fuel to the anode to cathode, possible this is for the small porous of the filler [29].

It is considered the methanol permeability normalized to the membranes thickness, the membranes with low SBA-15 load (S2 and S3) appear to have the better performance in terms of methanol permeation. When the inorganic loading increases up to 15%, the presence of filler does not produce a beneficial effect because the excessive amount causes a different arrangement of the polymeric structure resulting in higher permeability than other loadings. The S5 membrane (PWA/SBA-15) has even lower methanol permeability this could be attributed to a porous structured SBA-15 blocked by the PWA [29]. On the other hand, despite the low methanol permeability that S5 membrane presents, the selectivity, which is the ratio to the conductivity between the permeability (in S∙s∙cm^−3^) [38], due to the high conductivity of the S2 and S3 membranes, exhibits high selectivity to be used in DMFC. The S4 membrane shows the highest methanol permeability, due to, the high filler loading causes microporous, like SEM-EDS analyses show, resulting in a low selectivity.

In any case, since S-PEEK sulfonation degree has important effects on the mechanical properties as well as on its solubility, 5 wt.% and 10 wt.% SBA-15 amount, in S-PEEK with DS = 50%, seems to keep a good balance between the sulfonation degree and the physico-chemical characteristics of the filler.

### 3.3. Electrochmical Characterization

#### 3.3.1. Proton Exchange Membrane Fuel Cell

**Figure 7:**
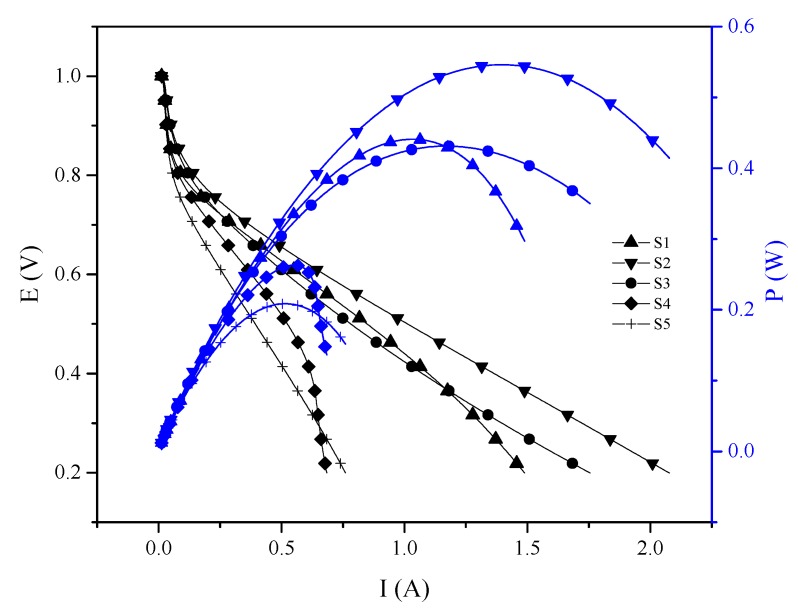
PEMFC Room temperature, P_abs_ = 1 atm, F_H2_ = 120 mL min^−1^, F_O2_ = 300 mL min^−1^, 100% RH.

In Figure 7 and Table 4, a comparison between the PEMFC performance and the ohmic resistance of the membranes measured during fuel cell tests at high frequency resistance (HFR) by Electrochemistry Impedance Scanning (EIS) are reported.

In a proton conducting membrane, the transport mechanism of contained species is related to its physico-chemical parameters and performance. The performance of S-PEEK with and without SBA-15, is attributed to the arrangement of the polymeric matrix as well as the different amount and acidity of sulfonic groups available depending on the inorganic load. In fact, S-PEEK has narrower and tortuous channels than Nafion^®^, resulting in a shorter path for the proton transport and, therefore, lower mobility and conductivity [26]. Higher IEC and Wup, with two negative consequences, mechanical instability and “dilution” of sulfonic groups, and then, lower proton conductivity [35,39]. For these reasons 50% sulfonation degree was chosen for this work, because it is enough to have an acceptable proton conductivity without losing mechanical stability during operation due to increase of water uptake. As expected, the resistance increases with SBA-15 loading, because the filler has no conductive functionality, so it is not convenient to use a loading higher than 10 wt.%.

The 10 wt.% SBA-15 (S3) membrane, despite the high resistance (0.412 ohm) shows a higher performance than S-PEEK (S1), 5 wt.% SBA-15 (S2) and 15 wt.% SBA-15 (S4). This behavior is coincident with the water uptake and λ at 30 °C. According to the XRD and BET results of the PWA/SBA-15 (S5), the heteropolyacid appears to be inside the SBA-15, therefore, its ability to drive protons is affected, which is reflected when the membrane is tested in PEMFC. Nevertheless, S2 membrane showed a total maximum power of 0.55 W (maximum power density, 110 W∙cm^−2^, compared to work Filippov et al. in where was studied the electro-diffusion characteristics of HNT-modified bilayer perfluorinated MF-4SC membranes, were similar maximum power density was found [20].

#### 3.3.2. Electrochemical Hydrogen Pumping

**Figure 8:**
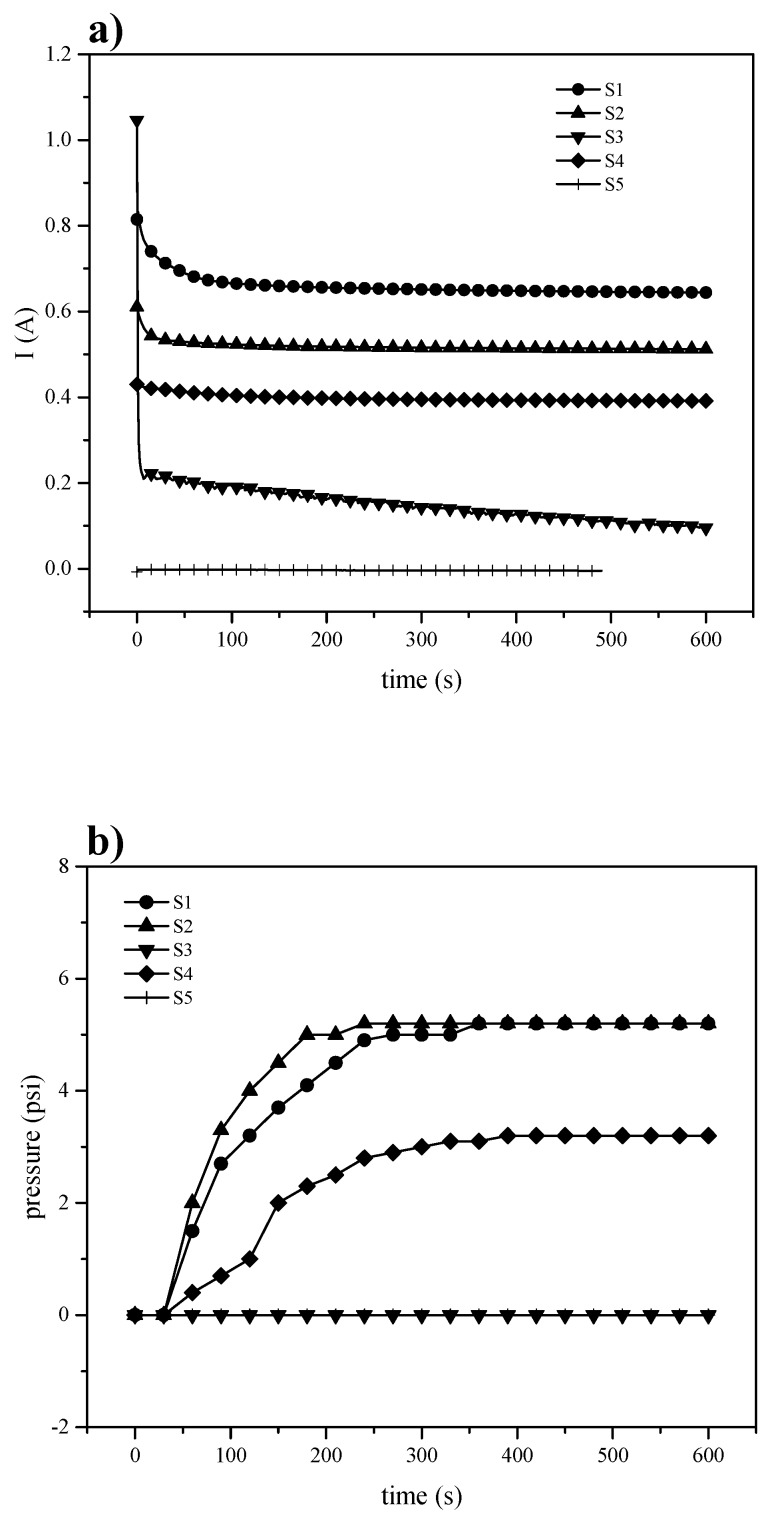
Electrochemical hydrogen pumping at 300 mV (**a**) current and (**b**) pressure.

The electrochemical hydrogen pumping aims to achieve gas pumping to conditions comparable to a mechanical compressor, but with lower energy consumption and without the production of pollutants. The proton exchange membrane in an EHC is subjected to high pressure difference between anode and cathode, and thus, it must be mechanically resistant. Since hydrogen reduction and oxidation reactions are kinetically fast in platinum as a catalyst, the EHC depends on the rate at which protons move through the membrane. In Figure 8a, it is shown the current intensity (left) associated to the hydrogen oxidation-reduction reactions on platinum at a potential pulse of 300 mV, and the pressure (Figure 8b right) measured in the pumping chamber (cathode). The S-PEEK membrane (S1), with its highest Wup, has higher performance than the others.

**Figure 9:**
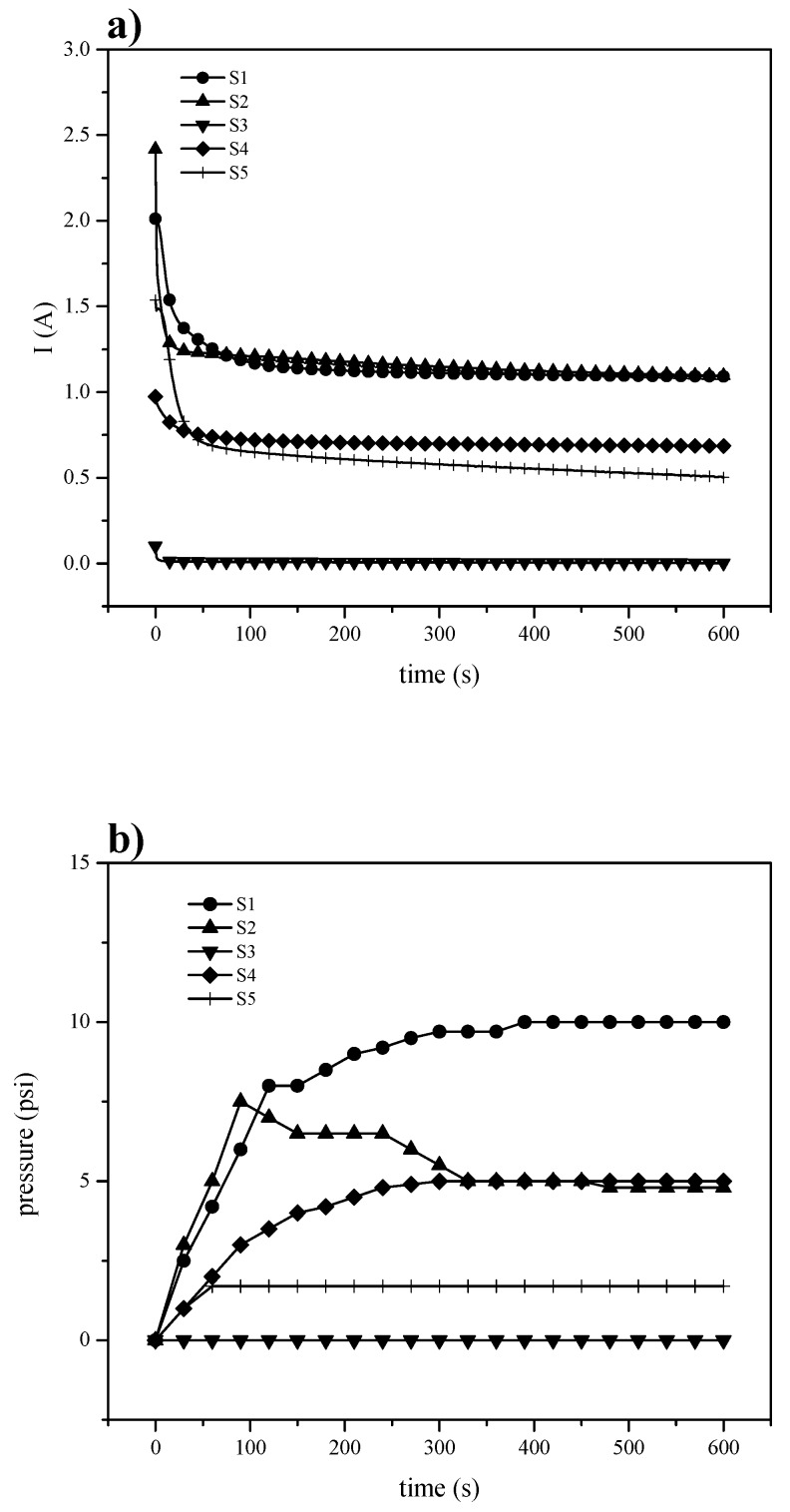
Electrochemical hydrogen pumping at 600 mV (**a**) current and (**b**) pressure.

The SBA-15 introduction could block the channels for proton transfer or even increase its tortuosity with the consequence of reducing the current intensity. Analyzing the pressure vs. time curves, it can be seen a similar behavior to that of current vs time, in fact, for the composite membranes the pressure decreases in accordance to the current intensity results. On the contrary, despite higher value of the current recorded for S-PEEK membrane, the corresponding pressure is lower than expected and comparable to S2 sample, probably due to a back-diffusion from cathode to anode, decreasing the membrane pumping capacity. This could mean that, in the presence of the inorganic filler, the blocking effect produces a beneficial effect in terms of pressure. Therefore, the increase of potential does not produce the same improvement in the pressure for the polymers (Figure 9), but S-PEEK response to the potential change is remarkably higher.

The Equation (7) relates the amount of hydrogen moles transferred per second once a steady state condition is reached, the highest current at the applied voltage (Table 5).

(7) $${\dot{N}}_{H_{2}}=\frac{I}{n\cdot F}$$

where I is the current in A, n is the number of transferred electrons (2e^−^ for this reaction), F is the Faraday constant (96485 C/e^−^), and ${\dot{N}}_{H_{2}}$ is the molar flux of hydrogen in mol s^−1^. All membrane electrode assemblies have the same electrode area; hydrogen flow was only normalized to the membrane thickness, proving that membrane structure as well as inorganic inclusion alters the proton transport and even leads to back-diffusion.

Although pumping efficiency is far from those reported by Ströbel [4] (8.1 psi cm^−2^) and Grigoriev [40] (28 psi cm^−2^), it should be mentioned that they used Nafion^®^ 117 and hardware specially designed as a compressor in order to withstand high pressure difference. Still, the characterization in this work allowed to foresee if S-PEEK with and without filler could be potentially used in an EHC.

## 4. Conclusions

Sulfonated polyether ether ketone (S-PEEK) was modified by the addition of SBA-15 and PWA previously supported on SBA-15, to prepare composite membranes as an alternative to Nafion^®^. These membranes were evaluated in three electrochemical energy systems, PEMFC, EHP. Although, the XRD pattern did not provide any evidence of the PWA presence in SBA-15, the lowering in surface area and pore diameter and the increase in IEC value could indicates a probable inclusion of PWA in SBA-15. In PEMFC, the membrane with 5 wt.% filler (S2), with its high λ and Wup, has an improving performance if compared to the other composites and the pristine membrane. In EHP, the inclusion of inorganic materials seems to increase the gas permeability causing back-diffusion from cathode to anode. However, the S2 membrane shown an improving time for the pressure increase in the cathode at 300 mV, raising the pressure at 5 psi in 180 s. For the methanol permeability test, the SBA-15 addition limits the methanol permeability. A 10 wt.% was found to be the better loading for this aim. Since each electrochemical device has different requirements, the development of a membrane able to properly perform in all applications here investigated is a not plausible task. In any case, composite membranes based on S-PEEK and 5% and 10% in SBA-15 seem to be promising for these applications, even if further investigations are necessary. Especially necessary is an improvement of the filler dispersion method.

## Figures and Tables

**Table 1:** Textural properties of inorganic fillers.

Inorganic Filler	Surface Area (m^2^/g)	Pore Diameter (nm)
SBA-15	782	9.4 ± 2
PWA	5	2.7 ± 1
PWA/SBA-15	289	7.7 ± 2
PWA/SBA-15 Washed	852	9.3 ± 2

**Table 2:** Values of water uptake, swelling, ion exchange capacity and ohmic resistance at 30 °C for several composite membranes.

Membrane	IEC	Wup %		Swelling		@30 °C
	meq g^−1^ (±0.097)	@30 °C (±2)	@80 °C (±3)	@30 °C (±0.15)	@80 °C (±0.2)	$\frac{mol\;H_{2}O}{mol\;SO_{3}H}$ (±2)
(S1) S-PEEK	1.63	46	79	1.2	1.5	16
(S2) S-PEEK+SBA15 5%	1.53	40	68	1.2	1.4	14
(S3) S-PEEK+SBA15 10%	1.36	45	58	1.3	1.3	18
(S4) S-PEEK+SBA15 15%	1.47	42	75	1.0	1.4	16
(S5) S-PEEK+SBA15 10% +PWA	1.42	40	52	1.0	1.2	16
Nafion 115	1.01	26	42	1.3	1.6	14

**Table 3:** Activation energies and pre-exponential factor for proton conduction from Arrhenius plots.

Membrane	Ea (eV)	σ_0_ (S cm^−1^)
S1	0.127	4.26
S2	0.130	4.76
S3	0.132	4.81
S4	0.199	8.05
S5	0.142	2.18

**Table 4:** ohmic resistance at 30 °C.

Membrane	R (Ohm) by EIS at HFR
S1	0.185
S2	0.251
S3	0.412
S4	0.6
S5	0.476

**Table 5:** Hydrogen transferred according to applied potential pulses.

Membrane	${\dot{N}}_{H_{2}}\;(\mathrm{mol}\;s^{-1})$ × 10^−6^		Pressure (psi)		Thickness (μm)	${\dot{N}}_{H_{2}}\;(\mathrm{mol}\;s^{-1}\;\mathrm{cm})$ × 10^−4^	
	300 mV	600 mV	300 mV	600 mV		300 mV	600 mV
S1	3.338	5.66	5.2	10	73	4.57	7.75
S2	2.654	5.67	5.2	7.5	80	3.32	7.09
S3	0.492	0.014	0	0	70	0.703	0.02
S4	2.03	3.554	3.2	5	90	2.26	3.95
S5	-	2.59	0	1.7	95	-	2.73
